# The clinical application of head-shaking test combined with head-shaking tilt suppression test in distinguishing between peripheral and central vertigo at bedside vs. examination room

**DOI:** 10.1016/j.bjorl.2022.03.003

**Published:** 2022-05-20

**Authors:** Huiying Sun, Yinping Wang, Hong Jiang, Zhiqiang Gao, Haiyan Wu

**Affiliations:** a Chinese Academy of Medical Sciences and Peking Union Medical College, Peking Union Medical College Hospital, Department of Otorhinolaryngology, Beijing, China; b Peking University, School of Public Health, Department of Maternal and Child Health, Beijing, China

**Keywords:** Head-shaking test, Head-shaking tilt suppression test, Central vertigo, Peripheral vertigo

## Abstract

**Objectives:**

To investigate the clinical value of using Head-Shaking Test (HST) + Head-Shaking Tilt Suppression Test (HSTST) to distinguish between peripheral and central vertigo as well as to analyze the consistency of findings between tests at the bedside vs. in the examination room.

**Methods:**

We retrospectively analyzed patients who presented for central or peripheral vertigo from July 2019 to July 2021. The results were compared between HST and HST + HSTST. The concordance between bedside and examination room outcomes was analyzed.

**Results:**

Forty-seven (58.8%) patients in the peripheral vertigo group and 33 (41.2%) patients in the central vertigo group were included. In the peripheral group, 44 (both examination room and bedside: 93.6%) patients had horizontal Head-Shaking Nystagmus (hHSN), most of which were suppressed in HSTST. However, in the central group, most cases had perverted HSN (pHSN; examination room: 72.7%; bedside: 66.7%), which was seldomly suppressed in HSTST. The HST + HSTST showed a >20% higher specificity in identifying peripheral vertigo than HST alone. The bedside results were consistent with the examination room results using the kappa test (*p* < 0.001).

**Conclusions:**

Suppressed hHSN was a strong indicator of peripheral vertigo. Conversely, pHSN was more often seen in central vertigo, which was not readily suppressed in HSTST. The bedside results of HST + HSTST yielded qualitative agreement with the tests in the examination room. HST + HSTST could be used as reliable methods in the clinic to distinguish between peripheral and central vestibular disorders.

**Level of evidence:**

Level 3.

## Highlights

• Combined HST and the HSTS test have a higher specificity than HST alone.
• Repressible horizontal HSN usually suggests a peripheral vertigo specifically.
• Findings of the bedside HST + HSTST are consistent with those of VNG.

## Introduction

Head-Shaking Nystagmus (HSN) is induced by rapidly shaking and immediately ceasing the head in the horizontal plane; it is a commonly used test to identify the asymmetry between the horizontal Vestibulo-Ocular Reflex (VOR). It is generally agreed that HSN is caused when the central velocity storage system, which includes vestibular nuclei, nucleus prepositus hypoglossi, nodulus and ventral uvula, releases low-frequency and low-velocity vestibular signals generated during the shaking motion.^1^ It has been reported that tilting forward may inhibit the horizontal HSN (hHSN). The possible mechanism behind this is that tilting forward excites the otolith organs, which elicit the signals in the direction of gravity^2^, ^3^, ^4^ thus inhibiting the velocity storage system and hHSN. The Head-Shaking Test (HST) and Head-Shaking Tilt Suppression Test (HSTST) can be used in discerning central and peripheral vertigo, respectively; however, their combined use to distinguish between peripheral and central vertigo has been seldom reported. HST and HSTST can be performed at the bedside and in the examination room. Whether the tests performed at the bedside are as reliable as those performed in the examination room needs discussion and support, and to our knowledge, this topic remains unexplored. This study was designed to investigate the clinical value of HST + HSTST in the identification of central and peripheral vertigo and to analyze the consistency of the results of the two tests at the bedside and in the examination room.

## Methods

### Participants

We retrospectively analyzed patients who presented with central or peripheral vertigo at the Balance Center, Department of Otolaryngology-Head and Neck Surgery in Peking Union Medical College Hospital from July 2019 to July 2021. The inclusion criteria were as follows: (1) Either peripheral or central vertigo, suspicious combination of peripheral and central vertigo was excluded by a series of neuroelectrophysiological examinations, physical examinations, and clinical symptoms; (2) Complete clinical data; (3) Completed HST and the HSTS test results at the bedside and in the examination room; and (4) HSN was elicited. The demographic characteristics, diagnosis, and results of HST and the HSTST at the bedside and in the examination, room were collected. The diagnosis was made according to the systematic evaluation of medical history, auditory tests, vestibular examinations, and radiological findings. All patients were divided into the peripheral vertigo group and central vertigo group. This study was exempted from the Institutional Review Board review by the Medical Ethics Committee of Peking Union Medical College Hospital owing to its retrospective design (S-K1760).

### HST and HSTST

For HST, patients were made to sit in the clinic room and instructed to wear a video goggle (SRM-PNG, Sireimei Medical Technologies Company, China). The portable goggle has the capacity to block visual fixation but cannot measure the slow-phase eye velocity. Their heads were inclined forward by 30° and rotated passively in the horizontal plane for 30 cycles at a speed of 2Hz – 3 Hz with the approximate amplitude of head-centered axes of ±10° by the same physician. Once they were sufficiently relaxed and the nystagmus disappeared, the patients undertook the HSTST. The head was rotated in the same way as in the HST; however, after rotation, patients were immediately instructed to tilt their head forward, rest their chin against the thorax. When the shaking stopped, the direction and intensity of the patient’s nystagmus were recorded until it ceased.

All patients were then made to wear a video nystagmograph (VNG, VisualEyes, system2000, Micromedical Technologies, USA) and HST and HSTST were reexamined. The direction and the velocity of the patient’s nystagmus were recorded using VNG. The examination room tests were finished by another physician.

HSN may be horizontal (hHSN), vertical, or combined (both horizontal and vertical). HHSN includes unidirectional and bidirectional subtypes. The bidirectional hHSN means that leftward and rightward nystagmus are introduced in succession after shaking the head, and the former nystagmus is recorded as phase I, while the latter is recorded as phase II. Vertical and combined nystagmus are defined as perverted HSN (pHSN), which shows vertical movements of the eyes while shaking the head horizontally.^5^

The Tilt Suppression Index (TSI) was calculated according to the velocity of the patient’s nystagmus as determined by VNG using the following formula: $\text{T}\text{S}\text{I}(\text{\%})=\frac{\text{s}\text{l}\text{o}\text{w}-\text{p}\text{h}\text{a}\text{s}\text{e}\;\text{e}\text{y}\text{e}\;\text{v}\text{e}\text{l}\text{o}\text{c}\text{i}\text{t}\text{y}\;\text{o}\text{f}\;\text{H}\text{S}\text{T}\text{S}\text{T}\;}{\text{s}\text{l}\text{o}\text{w}-\text{p}\text{h}\text{a}\text{s}\text{e}\;\text{e}\text{y}\text{e}\;\text{v}\text{e}\text{l}\text{o}\text{c}\text{i}\text{t}\text{y}\;\text{o}\text{f}\;\text{H}\text{S}\text{T}}\times 100$, where TSI < 80% was considered as inhibition, 80% ≤ TSI ≤ 120% was considered as no change, and TSI > 120% was considered as enhancement.^6^

### Statistical analysis

Data were analyzed using SPSS v. 23.0 (Chicago, IL, USA), and a *p* value of <0.05 (two-tailed) was considered to indicate statistical significance. Continuous variables that conformed to a normal distribution were reported as mean ± standard deviation and a parameter test was used. Data *that* did not conform to a normal *distribution* were reported as median (interquartile range) and a non-parametric test was used. Categorical variables were reported as number (percentage) and were analyzed using the χ^2^ or Fisher’s exact test. Receiver Operating Characteristic (ROC) curves were generated to analyze the sensitivity and specificity of HST and HSTST as screening tools at the bedside as well as in the examination room. The kappa statistic test was used to determine the concordance between bedside and examination room results, with a kappa value < 0.4 being inconsistent, and a kappa value ≥ 0.4 being consistent.

## Results

### Demographic characteristics

**Figure 1:**
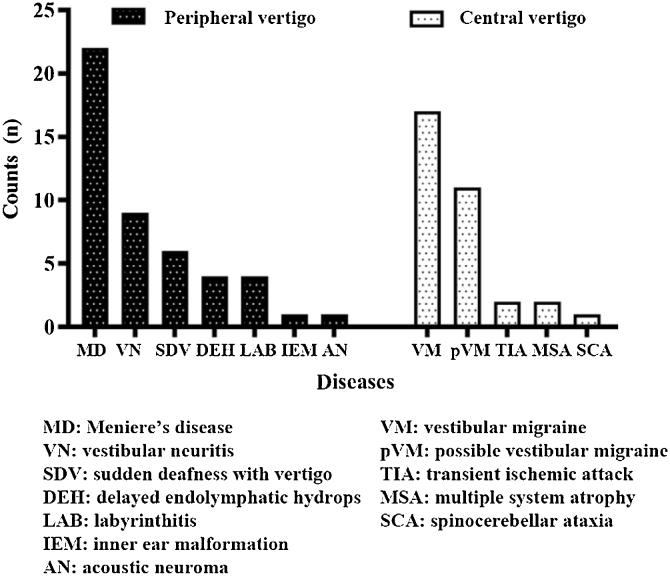
The diagnosis of patients in the peripheral vertigo group and central vertigo group.

A total of 80 patients (28 [35.0%] males, 52 [65.0%] females) were enrolled. Herein, 47 (58.8%) patients were in the peripheral vertigo group and 33 (41.2%) patients were in the central vertigo group. The average ages of peripheral and central vertigo group patients were 50.70 ± 13.26 years and 47.03 ± 16.65 years, respectively, with no significant difference (*p* = 0.276). The groups did not statistically significantly differ in terms of the sex of the patients (*p* = 0.460). The detailed information on diagnosis is shown in Fig. 1.

### HST and HSTST results by VNG

**Figure 2:**
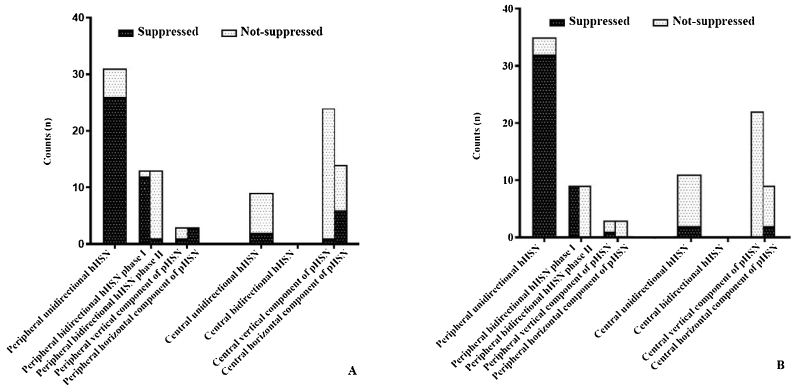
HST and HSTST results of peripheral vertigo group and central vertigo group. (A) Using VNG in the examination room; (B) Using a video goggle at the bedside.

In the peripheral vertigo group, 44 (93.6%) patients had hHSN, among whom 31 (70.5%) had unidirectional hHSN and the remaining has bidirectional HSN. Notably, HSTST inhibited unidirectional hHSN in 26 (83.9%) cases and phase I of bidirectional hHSN in 12 (92.3%) cases, and both showed significant decreases in the velocities of nystagmus by HSTST (both, *p* < 0.001). However, phase II of bidirectional hHSN was irrepressible in 12 (92.3%) cases. Only 3 (6.4%) cases had pHSN, and the horizontal component of pHSN was suppressed in all these cases (Fig. 2A**)**.

**Figure 3:**
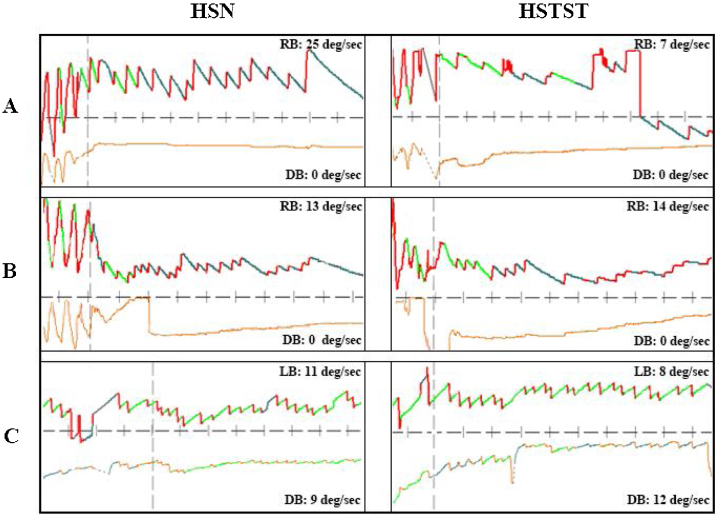
Three cases with peripheral or central vertigo. (A) A patient with peripheral vertigo: the hHSN was suppressed from 25°/s to 7°/s in HSTST, TSI = 28.0%. (B) A patient with central vertigo: the hHSN was not suppressed in HSTST, TSI = 107.7%. (C) Another patient with central vertigo: the horizontal component of pHSN was suppressed slightly; TSI = 72.7%, and the vertical component of pHSN was increased from 9°/s to 12°/s, TSI = 133.3%. TSI, Tilt Suppression Index; RB, Right Beat; LB, Left Beat; DB, Down Beat. The upper line represented horizontal nystagmus and the lower line represented vertical nystagmus.

In the central vertigo group, 9 (27.3%) patients had hHSN, and 24 (72.7%) had pHSN. Notably, hHSN was much more likely to occur in the peripheral vertigo group than in the central vertigo group (*p* < 0.001). The inhibition rate of horizontal eye movement by HSTST was much lower in the central vertigo group than in the peripheral vertigo group (*p* < 0.001). The velocities of hHSN and pHSN in the central vertigo group were not reduced by HSTST (*p* = 0.238; *p* = 0.073; *p* = 0.090, respectively) (Fig. 2A and Table 1). Fig. 3 shows one case each of peripheral and central vertigo groups.

**Table 1:** Comparison of slow-phase eye velocity of nystagmus between HST and HSTST by VNG in the examination room in the peripheral vertigo group and central vertigo group.

Slow-phase eye velocity of nystagmus (°/s)	Peripheral vertigo (n = 47)	*p*	Central vertigo (n = 33)	*p*
Horizontal				
Unidirectional				
HST	5.00 (3.00–10.00)	<0.001^a^	10.78 ± 8.12	0.238
HSTST	3.45 ± 3.80		7.78 ± 5.33	
Bidirectional			–	–
Phase I				
HST	10.62 ± 5.00	<0.001^a^		
HSTST	5.08 ± 4.63			
Phase II				
HST	5.23 ± 2.68	0.942		
HSTST	5.00 (3.00–6.00)			
Perverted				
Horizontal				
HST	11.00 ± 9.64	0.195	3.50 (2.75–5.25)	0.073
HSTST	7.00 ± 6.08		3.64 ± 3.15	–
Vertical				
HST	6.00 ± 2.65	0.902	5.13 ± 2.44	0.090
HSTST	5.67 ± 1.53		5.00 (4.00–7.25)	

HST, Head-Shaking Test; HSTST, Head-Shaking Tilt sSuppression Test; VNG, Video Nystagmograph.

a *p* < 0.05.

### HST and HSTST results at the bedside

The bedside results of HST + HSTST were quite similar to the results by VNG in the examination room. In the peripheral vertigo group, 35 (79.5%) patients had unidirectional hHSN, which could be suppressed by HSTST in 32 (91.4%) patients. Phase I of bidirectional hHSN was suppressed in all patients, whereas phase II could not be suppressed in any patient. In the central vertigo group, 11 (33.3%) patients had hHSN, which was irrepressible in 9 (77.8%) patients. The majority of the patients had pHSN (66.6%, 22 cases), which could not be suppressed by HSTST (Fig. 2B).

### HST vs. HST + HSTST

**Figure 4:**
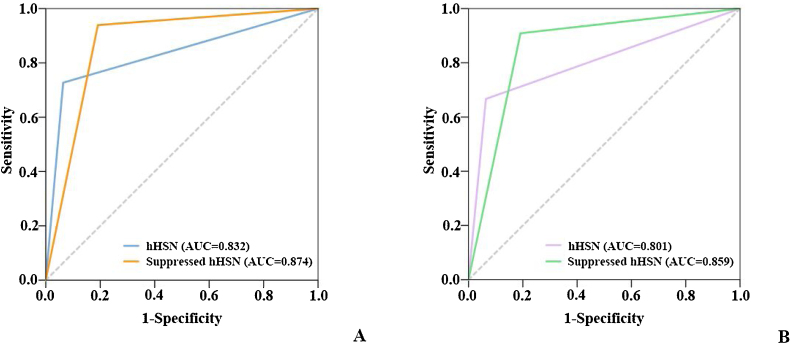
The ROC curves for HST only vs. HST + HSTST. (A) Using VNG in the examination room; (B) Using a video goggle at the bedside. AUC, Area Under Curve.

When VNG was used to screen for peripheral vertigo, the sensitivity and specificity of HST alone were 93.6% and 72.7% and those of HST + HSTST were 80.9% and 93.9%, respectively. The combination showed a >20% higher specificity than HST alone. The bedside results were in agreement with examination room results. The sensitivity declined because the criteria were stricter with the conditions of being horizontal as well as repressible (Table 2 and Fig. 4).

**Table 2:** Sensitivity and specificity of hHSN and repressible hHSN in distinguishing between peripheral vertigo and central vertigo.

	Examination room				Bedside			
	Peripheral vertigo, n (%)	Central vertigo, n (%)	Sensitivity (%)	Specificity (%)	Peripheral vertigo, n (%)	Central vertigo, n (%)	Sensitivity (%)	Specificity (%)
HST								
hHSN	44 (93.6)	9 (27.3)	93.6	72.7	44 (93.6)	11 (33.3)	93.6	66.7
pHSN	3 (6.4)	24 (72.7)			3 (6.4)	22 (66.7)		
HST + HSTST								
Repressible hHSN	38 (80.9)	2 (6.1)	80.9	93.9	41 (87.2)	3 (9.1)	87.2	90.9
Others	9 (19.1)	31 (93.9)			6 (12.8)	30 (90.1)		

hHSN, Horizontal Head-Shaking Nystagmus; pHSN, Perverted Head-Shaking Nystagmus; HST, Head-Shaking Test; HSTST, Head-Shaking Tilt Suppression Test.

### The consistency between bedside and examination room results

The bedside HST results were concordant with examination room results (kappa = 0.658, p < 0.001). The consistency between the results of HST + HSTST at the bedside and those of VNG was also confirmed by the kappa test (kappa = 0.650, p < 0.001) (Table 3).

**Table 3:** The consistency between the bedside and the examination room results.

HST	Examination room		Total	Kappa	*p*	HST + HSTST		Examination room		Total	Kappa	*p*
	hHSN (n)	pHSN (n)						Repressible hHSN (n)	Others (n)			
Bedside												
hHSN	48	7	55	0.658	<0.001	Bedside	Repressible hHSN	35	9	44	0.650	<0.001
pHSN	5	20	25				Others	5	31	36		
Total	53	27	80				Total	40	40	80		

HST, Head-Shaking Test; HSTST, Head-Shaking Tilt Suppression Test; hHSN, Horizontal Head-Shaking Nystagmus; pHSN, Perverted Head-Shaking Nystagmus.

## Discussion

HST is reportedly a useful, simple, and time-saving test for identifying vestibular disorders.^7^, ^8^, ^9^ HSN may be inhibited when the head tilts forward during HSTST.^10^, ^11^ HST and HSTST can be performed either at the bedside or in the examination room; however, to our knowledge, limited studies have reported the consistency between them. The findings of this study supported that the combination of HST and HSTST resulted in an improved specificity over HST alone in differential diagnoses of peripheral and central vertigo. Furthermore, this study assessed the concordance between HST + HSTS performed at the bedside and in the examination room.

In the present study, most HSN in the peripheral vertigo group was horizontal (both examination room and bedside: 93.6%), among which, a small proportion was bidirectional (examination room: 29.5%; bedside: 20.5%). The majority of unidirectional and bidirectional phase I hHSN could be suppressed by HSTST. However, phase II hHSN was rarely suppressed. This can be explained by the mechanism of HST and HSTST. As mentioned in the introduction, HSN is induced by the central velocity storage system which stimulates nystagmus in the shaking plane, which is in compliance with Ewald’s second law.^1^, ^12^ Thus, shaking head axially usually generates hHSN. Unidirectional hHSN often originates from acute unilateral peripheral defects, whereas bidirectional hHSN is widely considered an indicator of a transitional phase from acute to compensatory vertigo in unilateral peripheral defects. Phase II is believed to be the result of the compensation of the Central Nervous System (CNS) in response to the asymmetric input signals from bilateral vestibular organs.^7^ Tilting the head forward may excite the otolith organs in the gravitational direction but may not work on the compensatory mechanism.^13^ Therefore, unidirectional and phase I of bidirectional HSN can be easily suppressed, but phase II cannot be suppressed by HSTST.

In the central vertigo group, fewer patients (examination room: 27.3%; bedside: 33.3%) had hHSN compared with the peripheral vertigo group (*p* < 0.001), and bidirectional nystagmus was not observed in the central vertigo group. Higher incidence of pHSN was a characteristic of central group (examination room: 72.7%; bedside: 66.7%), which is in agreement with previous reports.^14^, ^15^, ^16^ Typically, pHSN has been observed in various diseases of the CNS, particularly in migrainous vertigo^17^, ^18^ and those closely associated with the cerebellum.^19^, ^20^, ^21^ There are two hypotheses about the mechanism of pHSN. (1) It could be attributed to incorrect connections and projections between the nucleus prepositus hypoglossi and the interstitial nucleus of Cajal, which participate in the horizontal and vertical VOR; (2) It could be attributed to the injured flocculus reducing the inhibition from the CNS to the superior semicircular canal, resulting in the enhanced input signals originating from the superior canal and leading to vertical nystagmus.^17^, ^22^, ^23^, ^24^ In the present study, the hHSN and pHSN were less susceptible to be inhibited in the central vertigo group than in the peripheral vertigo group (*p* < 0.001), concurring with the previous literature.^8^ Lesions damage the velocity storage system located in the brainstem and cerebellum may failed to be suppressed by HSTST as mentioned above.

It seemed that suppressed hHSN could specifically predict peripheral vertigo, whereas pHSN, even with HSTST, was not robust enough to identify central vertigo. Therefore, we studied the possibility of using suppressed hHSN to screen for peripheral vertigo. The solitary HST had high sensitivity but relatively low specificity in the differentiation of peripheral and central vestibular diseases. When used in combination with HSTST, the specificity of the HST was markedly improved by over 20%, indicating that HST + HSTST was more accurate in identifying peripheral vertigo. Considering the sensitivity of HST + HSTST was reduced by 13% compared with HST alone, the results of HST and HST + HSTST were recommended to considered together to complement each other, which was easily completed and applied in the clinic.

This study also compared bedside and examination room results, and consequently, the bedside outcomes were consistent with those of the VNG in the examination room (HST: kappa = 0.658, *p* < 0.001; HST+HSTST: kappa = 0.650, *p* < 0.001). The results suggested that bedside HST + HSTST is as capable as VNG in the examination room in identifying peripheral vertigo. The combination of HST and HSTST can be a useful and primary method in the differential diagnosis of vertigo in the clinic.

## Conclusion

HST + HSTST can be used to distinguish between peripheral and central vertigo, and suppressed hHSN often suggests a peripheral disease with a relatively high sensitivity and specificity. Furthermore, findings of HST + HSTST at the bedside have good consistency with those of VNG in the examination room, which provides preliminary information and reference for doctors to distinguish between peripheral and central vertigo in the clinic.

## Funding

This work was supported by Beijing Natural Science Foundation (7222313) and Key Program of Central HPB, National Health and Family Planning Commission of the People’s Republic of China (W2016ZD03).

## Availability of data and material

The datasets generated during and/or analyzed during the current study are available from the corresponding author on reasonable request.

## Ethics approval

This study was exempted from the Institutional Review Board (IRB) review by the Medical Ethics Committee of Peking Union Medical College Hospital (S-K1760).

## Conflicts of interest

The authors declare no conflicts of interest.
